# Placental DNA methylation at term reflects maternal serum levels of INHA and FN1, but not PAPPA, early in pregnancy

**DOI:** 10.1186/s12881-015-0257-z

**Published:** 2015-12-11

**Authors:** Samantha L. Wilson, John D. Blair, Kirsten Hogg, Sylvie Langlois, Peter von Dadelszen, Wendy P. Robinson

**Affiliations:** Child & Family Research Institute, 950 W 28th Ave, Vancouver, BC V5Z 4H4 Canada; Department of Medical Genetics, University of British Columbia, C201-4500 Oak St, Vancouver, BC V6H3N1 Canada; Department of Molecular & Cell Biology, University of California Berkeley, Berkeley, CA USA; Hudson Institute of Medical Research, Centre for Genetic Diseases, 27-31 Wright Street, Melbourne, Australia; Department of Obstetrics and Gynaecology, University of British Columbia, 4500 Oak St, Vancouver, BC V6H 3 V5 Canada

**Keywords:** Preeclampsia, Intrauterine growth restriction, DNA methylation, Maternal serum screening, Placenta

## Abstract

**Background:**

Early detection of pregnancies at risk of complications, such as intrauterine growth restriction (IUGR) and preeclampsia (PE), is critical for improved monitoring and preventative treatment to optimize health outcomes. We predict that levels of placental-derived proteins circulating in maternal blood reflect placental gene expression, which is associated with placental DNA methylation (DNAm) profiles. As such, placental DNAm profiling may be useful to distinguish pregnancies at risk of developing complications and correlation between DNAm and protein levels in maternal blood may give further evidence for a protein’s use as a biomarker. However, few studies investigate all clinical parameters that may influence DNAm and/or protein expression, which can significantly affect the relationship between these measures.

**Results:**

Candidate genes were chosen based on i) reported alterations of protein levels in maternal blood and ii) observed changes in placental DNAm (∆β > 0.05 and False Discovery Rate (FDR) <0.05) in pregnancies complicated by PE/IUGR. Fibronectin (*FN1*) enhancer DNAm and placental gene expression were inversely correlated (*r* = −0.88 p < 0.01). The same trend was observed between promoter DNAm and gene expression for *INHBA* and *PAPPA,* though not significant*. INHBA* and *FN1* DNAm was associated with gestational–age corrected birth weight, while INHA levels were associated with fetal: placental weight ratio and FN1 level was associated with maternal body mass index (BMI).

DNAm at the *INHBA* promoter in the term placenta was negatively correlated with second trimester maternal serum levels (*r* = −0.50 *p* = 0.01) and DNAm at the *FN1* enhancer was negatively associated with third trimester maternal serum levels (*r* = −0.38, *p* = 0.009). However, a similar correlation was not found for *PAPPA*.

**Conclusions:**

These results show that establishing a correlation between altered DNAm in the term placenta and altered maternal serum levels of the corresponding protein, is affected by a number of factors. Nonetheless, the correlation between placental DNAm of *INHBA*/*FN1* and maternal serum INHA/FN1 levels indicate that DNAm may be a useful tool to identify novel biomarkers for adverse pregnancy outcomes in some cases.

**Electronic supplementary material:**

The online version of this article (doi:10.1186/s12881-015-0257-z) contains supplementary material, which is available to authorized users.

## Background

Placental insufficiency is the inability of the placenta to provide an adequate supply of nutrients to the growing fetus. This can lead to a number of pregnancy complications including intrauterine growth restriction (IUGR) [1] and preeclampsia (PE), a maternal hypertensive disorder, which manifests as maternal hypertension and proteinuria after 20 weeks (wks) gestation [2]. Early diagnosis of PE and IUGR before clinical signs of disease can improve management and outcomes of affected pregnancies. Placental-derived proteins may be released into the maternal circulation where they can be quantified and used to assess placental function during pregnancy [3–6]. Such protein markers have been investigated for the prediction of PE and/or IUGR with varying success [7–9]. Nicolaides *et al.* (2013) reported a detection rate of 95 % for early-onset PE (EOPE, diagnosis <34 wks) using decreased levels of maternal serum markers, pregnancy associated plasma protein A (PAPPA) and placental growth factor (PlGF), in combination with maternal factors [7]. However, these measures might not be generalizable, as the etiology and confounding environmental factors vary between populations [8]. Moreover, the ability to predict women at risk of late-onset PE (LOPE, diagnosis >34 wks) and IUGR is limited using these markers.

Differential gene expression between placentas from PE and/or IUGR pregnancies [9–12] may be utilized to identify additional biomarkers to distinguish women at high risk of these complications early in gestation. DNA methylation (DNAm) is associated with gene expression, but is more robust to variation in technical conditions and less subject to short-term biological change [13]. We previously reported numerous changes in DNAm in placentas from pregnancies complicated by EOPE [14]. Alterations of placental DNAm were noted in genes for which the expression of the encoded protein is altered in maternal blood in PE and/or IUGR pregnancies (e.g.: *PAPPA, sENG, PAPPA2*) [14]. Furthermore we found that sites of altered DNAm in PE frequently reflected changes in gene expression. While proteins produced in the placenta can be released into maternal circulation, their levels in maternal serum may be affected by many additional factors including size of the placenta, the cell type expressing the protein, and how such proteins are transported and metabolized. The purpose of the present study was to delineate the relationship between changes we observed in DNAm at term and maternal protein levels in early pregnancy. We selected three genes for which there was evidence for both altered maternal protein levels and altered DNAm in PE; we then evaluated 1) the relationship between placental DNAm and gene expression; 2) the role of variables that might confound measurement of DNAm, mRNA or protein levels including gestational age, fetal sex, placental efficiency (fetal: placental weight ratio), fetal birth weight, placental breadth: width ratio and maternal body mass index (BMI); and 3) whether placental DNAm at term reflected protein levels in maternal blood during gestation after correcting for these variables.

## Results and discussion

### Candidate site selection and characteristics

To isolate loci for which altered DNAm might reflect maternal serum levels early in pregnancy, we chose candidate genes that not only had sites showing altered DNAm in EOPE, but also encode for proteins previously reported to show altered maternal serum protein levels in pregnancies that subsequently developed PE and/or IUGR. Previous studies have shown upregulation of both *PAPPA* and *INBHA* in the placentas of pregnancies complicated by PE and IUGR [12, 14–16]. In addition, several studies have reported DNAm alterations in placentas from pregnancies complicated by PE and/or IUGR [14, 17, 18]. *FN1* [19] was selected due to the large magnitude of change in DNAm between EOPE and control placentas (∆β = −0.24, FDR < 0.05) (see methods). *INHBA (*∆β = −0.16, FDR < 0.05) and *PAPPA (*∆β = −0.074, FDR < 0.05) were selected because they additionally encode for proteins for which first (PAPPA) or second trimester (INHA) maternal serum measures were available from clinical prenatal serum screening testing. We also focused on DNAm alterations in gene regulatory elements. The CpGs of interest for *INHBA* and *PAPPA* were 76 base pairs (bp) and 163 bp upstream of the transcriptional start sites, respectively. In relation to *FN1*, the CpG site was ~100 kb upstream of the transcriptional start site, within an enhancer region.

**Fig. 1:**
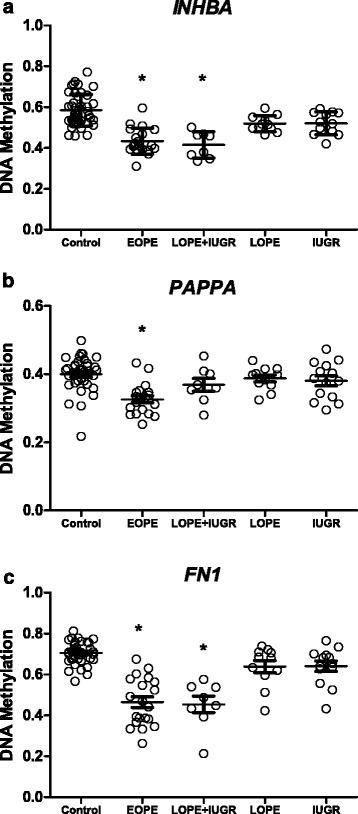
DNAm distribution at *INHBA*, *PAPPA*, and *FN1* across all clinical groups. The DNAm distribution (β values ± SD) at each site across clinical groups for **a** *INHBA*, **b** *PAPPA*, and **c** *FN1*. EOPE = early-onset PE (*N* = 20), LOPE = late-onset PE (*N* = 11), IUGR = Intrauterine growth restriction (N = 12), Control (N = 37). *p < 0.05

Although these sites were selected based on a significant association with EOPE, we also wanted to know if these changes were conserved in other clinical groups (Fig. 1). In addition to hypomethylation of these sites in EOPE, the LOPE + IUGR group was hypomethylated for the *INHBA* (promoter) (∆β = −0.18, *p* < 0.001) (Fig. 1a) and the *FN1* upstream enhancer (∆β = −0.25, *p* < 0.01) (Fig. 1c). While reduced methylation at the *PAPPA* promoter was only found in EOPE (Fig. 1b). As differences in DNAm were only found in the EOPE and LOPE + IUGR groups, as potential biomarkers, these candidate genes would presumably only be useful in identifying this subset of pregnancies [20]. Markers useful to detect LOPE or normotensive IUGR may be more challenging to identify due to their weak association with placental pathology.

### Is DNAm at candidate sites inversely correlated with gene expression?

**Fig. 2:**
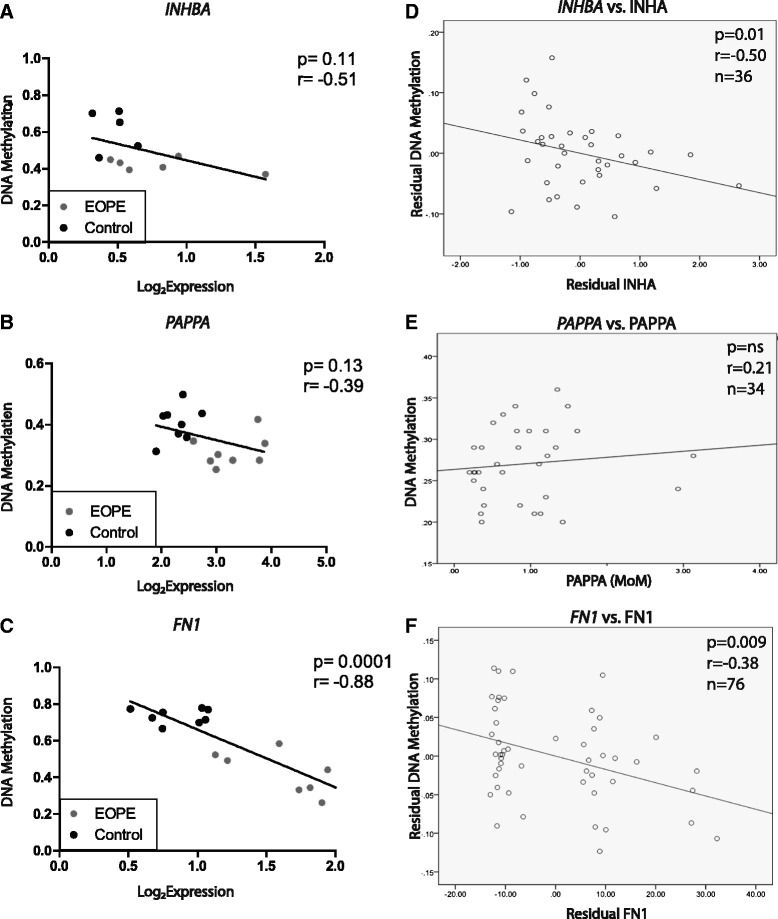
Correlation between placental DNAm and gene expression at term in control samples and between placental DNAm and maternal blood protein levels during gestation in control samples. The correlation between DNAm at a regulatory element and gene expression (log2) in eight early-onset PE and eight control placentae in **a** *INHBA* **b** *PAPPA* and **c** *FN1* † All gene expression graphs were produced from data published in Blair *et al.* (2013). The relationship between **d** *INHBA* (*N* = 36) promoter DNAm in the term placenta and second trimester INHA levels in maternal blood, plotted as residuals corrected for fetal birth weight (SD) and fetal: placental ratio, **e** *PAPPA* (*N* = 34) promoter DNAm in the term placenta and first trimester PAPPA levels in maternal blood, and **f** *FN1*(*N* = 76) enhancer DNAm in the term placenta and second/third trimester FN1 levels in maternal blood, plotted as residuals corrected for fetal birth weight (SD), gestational age, and maternal body mass index (BMI). MoM = multiple of the median

To confirm that the DNAm change resulted in a change in gene expression, we assessed the relationship between placental DNAm (measured by Illumina 450 k array) and gene expression at these three candidate sites. *FN1* showed an inverse correlation between DNAm of an upstream enhancer and gene expression at term (*r* = −0.88, *p* < 0.0001). *INHBA* and *PAPPA*, showed a non-significant trend with increasing DNAm being associated with decreased gene expression in the placenta (Fig. 2). This phenomenon may be due to alterations in cell composition between pathogenic and healthy placentas related to the pathology of PE/IUGR [14]. For all candidate genes, there was an observable divide between the controls and EOPE cases, where cases had decreased DNAm corresponding to increased gene expression in the placenta.

### What clinical factors are associated with DNAm at candidate sites?

To better understand what factors might affect the measurement of DNAm and therefore the relationship with protein expression levels in maternal blood, we also evaluated several potential confounding factors including gestational age at delivery [21], fetal sex [22, 23], fetal birth weight [24], placental dimensions and maternal BMI. Bisulfite pyrosequencing was used to extend our assessment of DNAm at the candidate sites into a larger cohort of controls for which clinical serum measurements (*INHBA N* = 36, *PAPPA N* = 33) or serum samples for assaying *FN1* (*N* = 76) were available.

Birth-weight standard deviation (SD) was associated with DNAm at the *INHBA* promoter (*p* = 0.05) and the upstream enhancer of *FN1* (*p* = 0.02). Gestational age was only associated with *FN1* DNAm (*p* = 0.03). None of the clinical factors assessed was associated with DNAm at the *PAPPA* site (Table 1). The observation that birth weight (SD) was associated with *INHBA* DNAm, without an association with gestational age, emphasizes the importance of including both gestational age and birth weight when considering the relationship between DNAm and other variables.

**Table 1:** Univariate linear analysis results (DNAm vs. Clinical parameters) in controls. Reported in correlation coefficient (r) values

Gene	N=	Fetal Sex	GA at Delivery	Birth Weight (SD)	Fetal: Placental Weight	Placental	Maternal BMI
						Length: Breadth	(Number of samples BMI was available)
*INHBA*	36	0.53	0.055	0.29*	0.08	0.01	0.35 (*N* = 18)
*PAPPA*	34	0.18	0.17	0.24	0.00	0.26	0.25 (*N* = 21)
*FN1*	76	0.12	0.22*	0.23*	0.10	0.23	0.30 (*N* = 75, all samples)
							0.12 (*N* = 37,control only)

GA gestational age

*p < 0.05

### What clinical factors are associated with protein concentration in maternal blood?

We also assessed the same clinical parameters for association to protein concentration in maternal blood (Table 2). Gestational age at blood draw was only assessed as a covariate for FN1 as clinical values for INHA and PAPPA were given in multiples of the median (MoM), which was already corrected for GA at blood draw. Placental efficiency (fetal: placental weight-ratio, at birth) was associated with increased second trimester INHA levels in maternal blood. FN1 level was not associated with maternal BMI in the controls for which we had this information (*N* = 37), though it was significant when evaluating all clinical groups together (EOPE, LOPE, IUGR, Controls) (*N* = 75). It was therefore included in subsequent analyses. None of the assessed factors were associated with PAPPA maternal blood levels during pregnancy (Table 3).

**Table 2:** Samples used for pyrosequencing and to assess maternal FN1 protein levels

	Control	EOPE	LOPE + IUGR	LOPE	IUGR
INHA N=	36	-	-	-	-
Mean GA at blood draw (weeks ± SD)	14–20wks	-	-	-	-
Mean GA at delivery (weeks ± SD)	39.3 (±1.3)	-	-	-	-
Mean BW (grams ± SD)	3480.3 (±483.4)	-	-	-	-
Mean MA (years ± SD)	33.5 (±4.4)	-	-	-	-
Sex (Female/N, %)	18/36, 50 %	-	-	-	-
PAPPA N=	33	-	-	-	-
Mean GA at blood draw(weeks ± SD)	11–13wks	-	-	-	-
Mean GA at delivery (weeks ± SD)	39.6 (±1.4)	-	-	-	-
Mean BW (grams ± SD)	3428.9 (±355.9)	-	-	-	-
Mean MA (years ± SD)	34.2 (±4.6)	-	-	-	-
Sex (Female/N, %)	18/34, 53 %	-	-	-	-
FN1 N=	76	13	6	10	9
Mean GA at blood draw(weeks ± SD)	31.6 (±6.1)	32.3 (±3.2)	35.9 (±1.3)	37.4 (±2.4)	33.5 (±4.5)
Mean GA at delivery (weeks ± SD)	39.1 (±2.9)	33.1 (±3.2)	36.1 (±1.1)	38.4 (±1.9)	35.2 (±4.5)
Mean BW (grams ± SD)	3465.3 (±398.94)	1663 (±710)	1921 (±402)	3187 (±683)	1932 (±746)
Mean MA (years ± SD)	33.5 (±3.6)	33.4 (±6.4)	32.4 (±5.3)	35.5 (5.5)	33.5 (±3.5)
Sex (Female/N, %)	^a^36/74, 49 %	6/13,46 %	3/6, 50 %	6/10, 60 %	6/9,66 %

^a^Sex not available on 2 samples

**Table 3:** Univariate linear analysis results (Protein Levels vs. Clinical parameters) in controls. Reported in correlation coefficient (r) values

Protein	*N*=	Fetal Sex	GA at Delivery	GA at Blood Draw^a^	Birth Weight (SD)	Fetal Weight: Placental Weight	Placental Length:Breadth	Maternal BMI (Number of samples BMI was available)
INHA	36	0.20	0.00	NA	0.12	0.44*	0.30	0.34 (*N* = 18)
PAPPA	34	0.11	0.20	NA	0.26	0.08	0.00	0.05 (*N* = 21)
FN1	76	0.05	0.10	0.16	0.11	0.13	0.063	0.25* (*N* = 75, all samples)
								0.10 (*N* = 37, control only

GA gestational age

*p < 0.05

^a^Only measured for FN1 as INHA and PAPPA levels were obtained from maternal serum screening program and already corrected for gestational age at blood draw

### What is the relationship between DNAm and maternal serum levels?

DNAm in the promoter of *INHBA* correlated with second trimester protein levels in maternal blood (*r* = −0.50, *p* = 0.01) while modeling for both fetal birth weight (SD) and fetal: placental weight ratio (Fig. 2d). Additionally, DNAm in an upstream enhancer of *FN1* correlated with third trimester protein levels in maternal blood (*r* = −0.38, *p* = 0.009) while adjusting for birth weight (SD), gestational age, and maternal BMI (Fig. 2f). This supported our prediction that DNAm changes observed in the placenta could explain some of the previous reports of altered INHA and FN1 levels in maternal blood in PE. It is remarkable that these serum measurements from the second and third trimesters of pregnancy reflected DNAm at term. This implies that this DNAm change may be an early alteration in PE. In contrast, a similar result was not observed for *PAPPA*/PAPPA (Fig. 2e).

**Fig. 3:**
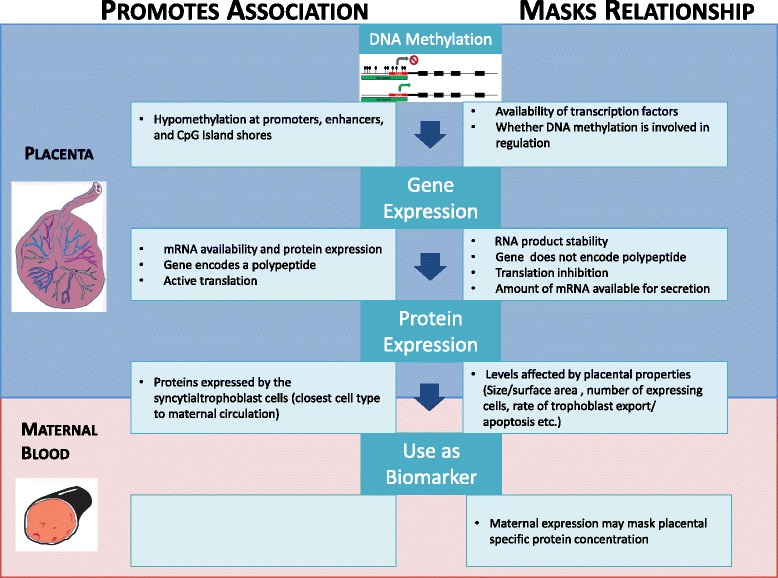
Processes that may influence the relationship between DNAm, gene expression and protein expression. Outlines reasons why we may not see a correlation between placental DNAm and gene expression or between placental gene expression and circulating levels of placental-specific proteins in maternal blood

We had predicted that protein levels in maternal blood would reflect placental DNAm and gene expression. While this may be true in some instances (e.g. INHA, FN1), in other cases establishing a relationship may be challenging (e.g. PAPPA). Establishing such a relationship may be complicated by several factors. Protein level depends not only on the level of gene expression, but also on the total number of cells expressing that protein, the number of mRNA transcripts being translated into protein in those cells, and the rate and mode of release of the protein into maternal blood. These factors may be influenced by the underlying pathology (i.e. more protein may be released with increased apoptosis) and placental size; which, in turn may be associated with fetal weight and/or fetal: placental weight ratio. Other factors such as expression of the same protein from maternal tissues, and the metabolism of proteins by the placenta, reducing the amount of protein being secreted into the maternal circulation may have a substantial influence of the total protein concentration in maternal blood (Fig. 3). PAPPA has been found to be expressed from other maternal sources (e.g. ovary, some epithelial and endometrial cells, and breast) besides the placenta, and it is possible be that these sources mask any relationship between placental derived protein and DNAm in the placenta [25–28]. It is also important in the case of PAPPA to note that maternal protein levels were measured in the first trimester and additional variation may arise over gestation affecting correlation with placental DNAm at term.

### Are there any differences in protein levels between case and control placentas?

**Fig. 4:**
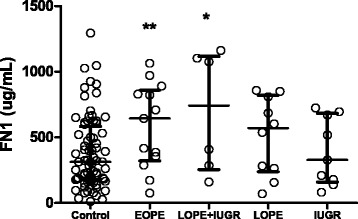
FN1 protein levels in maternal blood during gestation across all clinical groups. FN1 levels (Median with interquartile range) in maternal blood are increased in EOPE compared with controls, with a increasing trend in LOPE + IUGR compared to controls. EOPE = early-onset PE (*N* = 20), LOPE = late-onset PE (*N* = 11), IUGR = Intrauterine growth restriction (*N* = 12), Control (*N* = 37). **p < 0.05, *p < 0.1

To confirm a previous report of altered maternal FN1 in association with PE and/or IUGR [19], FN1 levels were measured in maternal blood samples from pregnancies which subsequently developed EOPE, LOPE + IUGR, LOPE without IUGR, or normotensive IUGR, in addition to our control cohort (Table 2). Similar to the alterations in DNAm, changes in FN1 levels were found to be significantly different from controls only in the EOPE group (Mann U Whitney test), although there was a trend of increased FN1 levels between LOPE + IUGR and controls (*p* = 0.08) (Fig. 4). Our results were in concordance to Auer et al. (2010) who also reported increased levels of maternal FN1 in pregnancies complicated by EOPE and LOPE + IUGR. We did not confirm their observation of a decrease of FN1 in pregnancies complicated by IUGR; however, we may have been under-powered to observe this small difference. Furthermore, although we observe a difference in EOPE and LOPE + IUGR compared to controls, the range of FN1 levels completely overlap between the groups, hindering FN1 to be an adequate biomarker used alone.

## Conclusion

This study provides a link between changes in placental DNAm at term and protein biomarkers present in the mother’s circulation earlier in pregnancy. It emphasizes the many confounding factors that may influence this relationship, explaining why this linkage may not be observed for all loci. We chose three genomic sites with significantly altered DNAm in term placenta associated with PE and that were associated with genes for which the protein product is altered in PE/IGUR. Despite this, for only two of the three loci *(INHA* and *FN1*) did we find a correlation between placental DNAm and second and third trimester maternal serum protein expression in control samples. Nonetheless, this does suggest that other DNAm marks may be associated with early differences in gene expression. Furthermore, with the advent of techniques to quantify placental nucleic acids in maternal serum [29], DNAm changes may be more directly linked to measurable miRNA and RNA in maternal blood. Factors such as placental surface area and mechanisms for release into maternal blood, will also affect serum levels of placental nucleic acids [30]. Future studies measuring protein levels directly in placental tissue, correlating with maternal levels and investigating the factors affecting rate of release are needed to help translate findings measured in the term placenta into maternal biomarkers of pregnancy outcomes in early gestation.

## Methods

### Sample information

Ethics approval was obtained from both the University of British Columbia and BC Women’s and Children’s Hospital ethics committees in Vancouver, BC, Canada (H04-70488). Placental samples were obtained with consent via recruitment through the Medical Genetics and Obstetrics and Gynecology departments. Case information such as: maternal age, maternal BMI, mode of delivery, gestational age at delivery, fetal sex, birth weight, gestational age at blood draw, results on any molecular testing, and placental dimensions were recorded.

Preeclampsia (PE) was defined according to Society of Obstetricians and Gynecologists of Canada (SOGC) criteria as one of i) hypertension (BP > 140/90 mm Hg) and proteinuria (>300 g/day) arising after 20 weeks gestation [2]; ii) HELLP syndrome without hypertension or proteinuria [31]; or iii) eclamptic seizure without previous hypertension or proteinuria [32]. EOPE was defined by a diagnosis of PE prior to 34 weeks gestation, and LOPE was defined as a diagnosis after 34 weeks gestation [33]. Intrauterine growth restriction (IUGR) was also defined following SOGC criteria [34] as birth weight < 3rd percentile accounting for fetal sex and gestational age, or birth weight < 10th percentile with additional clinical findings indicative of poor growth such as: absent or reversed end diastolic velocity on Doppler ultrasound, or oligohydramnios. Criteria for exclusion were chronic/pre-existing maternal hypertension, gestational diabetes, multi-fetal pregnancies, and fetal chromosomal abnormalities. Controls were selected based on absence of any criteria listed above and a placenta with no observable pathology.

Whole chorionic villi were sampled from four sites, each from distinct cotelydons of the placenta [13]. Sampling from infarcts or other abnormal regions of the placenta was avoided. DNA was extracted from each sampled site and pooled together in equal proportions. DNA was assessed for quality on the Nanodrop 1000 spectrophotometer (ThermoScientific, Wilmington, DE, USA). Three hundred nanograms of each DNA sample was bisulfite converted for subsequent analyses. Additionally, RNA extracted from the placental villi with RNeasy kit (Qiagen, Heiden, Germany) and was stored in RNAlater at−80 °C. RNA quality was assessed on a Bioanalyzer 2100 (Agilent, Santa Clara, USA).

While we used a total of 171 placentas for our studies, not all placentas were used in all studies as we were limited by samples run on the 450 K array (*N* = 66); samples run on the Illumina expression array (*N* = 16), maternal serum screening results (first trimester *N* = 34, second trimester *N* = 36), or maternal serum samples for FN1 testing (*N* = 114). Additional file 1: Table S1 outlines a list of all samples and which analyses they were used in.

### Gene expression analysis

Gene expression was measured with the HT-12v4 Expression BeadChip (Illumina, Inc.) as per Blair et al. (2013) protocol, comparing eight EOPE and eight controls [14] (Additional file 2: Table S2).

### DNA methylation analysis

#### Illumina infinium HumanMethylation450 BeadChip array

To compare the DNAm differences between clinical groups for each of our candidate genes twenty EOPE, 11 LOPE, 8 LOPE + IUGR, 10 IUGR, and 37 control cases were run on the Illumina Infinium HumanMethylation450 BeadChip (450 k) array, which interrogates >480,000 CpG sites in >20,000 genes [35]. Some of these samples were previously analyzed in the study reported by Blair et al. (2013). To compare the association between DNAm and protein levels in maternal blood, 122 placental DNA samples (750 ng) bisulfite converted using the EZ DNA Methylation kit (Zymo Research, Irvine, USA). Hybridization of samples to the array was completed as per the manufacturer’s protocol. The microarray chips were scanned by the HiScan 2000 or iScan (Illumina). Data was normalized and analyzed as per Blair *et al.* (2010) methods [14].

#### Bisulfite pyrosequencing

Candidate CpGs determined from the 450 k array data in Blair *et al.* (2013) were followed up with bisulfite pyrosequencing in control cohorts for each candidate gene (Table 4). To compare the association between DNAm and protein levels in maternal blood, 122 placental DNA samples (750 ng) were bisulfite converted using the EZ DNA methylation-Gold kit (Zymo Research Corp, Irvine, CA, USA) as per manufacturer’s protocol. Bisulfite converted DNA was PCR amplified prior to pyrosequencing. PCR reactions consisted of 20 ng of bisulfite converted DNA, 1x PCR buffer (with MgCl_2_) (Qiagen Ltd.), 0.18U DNA polymerase (HotStarTaq, Qiagen Ltd.),0.2 mM dNTP (Invitrogen, Carlsbed, CA),0.4uM forward and reverse primers (Integrated DNA Technologies, Coralville,IA) for *INHABA,PAPPA,* and *FN1*. PCR conditions were 95 °C (15 min), [95 °C (30s), 55 °C (30s), 72 °C (30s)]x40 cycles, 72 °C (10 min). Pyrosequencing assays for the candidate genes were designed in PSQ Assay Design software (Biotage, Upsalsa, Sweden) and run on a Qiagen Pyromark Q96 MD (Qiagen) (Additional file 3: Table S3).

**Table 4:** Candidate CpG sites chosen for follow-up

Gene	Site	Genomic Region	Distance to TSS (bp)	EOPE (Change in Beta value from control group)
*INHBA*	cg11079619	Active Promoter	76	0.434 (−0.162)
*PAPPA*	cg08189448	Active Promoter	−163	0.326 (−0.074)
*FN1*	cg12436772	Intergenic/Upstream enhancer	−101593	0.465 (−0.240)

#### Candidate DNAm selection

CpG sites chosen to investigate in the present study were selected on i) a significant change in placental DNAm, defined as a false discovery rate (FDR) < 0.05 a ∆ β > 0.05 (i.e. at least 5 percentage points difference in DNAm), a cut-off that enriches for changes in DNAm that would likely have biological impact [36], in placentas associated with PE and ii) genes encoding for proteins reported to show altered levels in maternal blood in pregnancies complicated by PE and/or IUGR. In addition to meeting these criteria, *INHBA* and *PAPPA* were chosen as we had maternal serum measures available on INHA and PAPPA from the maternal serum-screening program. We chose FN1 since the difference in DNAm between EOPE and controls was Δβ = 0.24. We also took into account where the DNAm alteration was in the genome, taking interest in alterations in gene regulatory elements (Table 4).

#### Maternal blood protein measurements

Measurements of Pregnancy associated plasma protein A (PAPPA) and Inhibin alpha (INHA) were obtained from clinical maternal serum screening data for 36 and 33 women, respectively, and are measured in multiples of the median (MoM). Additionally, blood was drawn in EDTA tubes during the second trimester for a subset of 158 women (Table 2). Plasma was obtained via centrifugation at 3000 rpm for 10 min 4 °C. Plasma Fibronectin (FN1) was measured using a FN1 ELISA kit (eBioscience, San Diego, CA, USA). FN1 measurements were run in duplicate and absorbance was measured at 450 nm. A 5 parameter asymmetrical logistic curve was generated from the standard data points which ranged from 0.31-20.0 ng/mL. Samples were diluted as per manufacturer’s protocol; samples which FN1 concentration was over the standard curve were diluted to 1 in 80,000, and 4 samples which remained were further diluted to 1 in 100,000.

#### Statistical analysis

DNAm at the two CpGs in the *PAPPA* pyrosequencing assay were correlated (*r* = 0.85, *p* < 0.001, Spearman’s correlation) and the measurements for these two sites were thus averaged (Additional file 4: Figure S1).

Potential covariates which may be associated with either DNAm or protein concentration in maternal blood were assessed for each candidate site. Univariate linear regression analyses were performed, investigating gestational age at delivery, fetal sex, fetal birth weight (SD), fetal: placental weight ratio, placental length: breadth ratio, maternal BMI, and when appropriate, gestational age at blood draw. As absolute fetal birth weight is confounded by gestational age at delivery, fetal birth weight was measured as a standard deviation relative to the mean for that gestational age. PAPPA and INHA protein levels were expressed in MoM to correct for gestational age a blood draw.

Correlations were performed when testing any association between placental gene expression at term and placental DNAm at term. Spearman’s correlations were performed between protein concentration and DNAm in sites where there were no covariate factors. For sites with covariate factors, which needed to be modeled for, partial correlations were performed. Non-parametric t-tests were performed to determine if DNAm in the EOPE, LOPE + IUGR, LOPE, and IUGR placentas were significantly different from controls. Statistics were calculated using SPSS v19.0 statistical package.

## Additional files

Additional file 1: Table S1. All samples used and the measures completed on each one. (PDF 353 kb) Additional file 2: Table S2. Information on samples used to assess DNAm and gene expression in the placenta. (PDF 174 kb) Additional file 3: Table S3. Primer sequences for bisulfite pyrosequencing. Specific locations are based on UCSC hg/18 assembly. (PDF 296 kb) Additional file 4: Figure S1. Spearman’s correlation between CpG 1 and CpG 2 within the *PAPPA* assay. DNA methylation was averaged over the two CpGs (PDF 249 kb)

## Abbreviations

450 k array: Illumina HumanMethylation450 bead chip array.
DNAm: DNA methylation
EOPE: Early-onset preeclampsia
FN1: Fibronectin
INHBA: Inhibin beta-alpha
IUGR: Intrauterine growth restriction
LOPE: Late-onset preeclampsia
PAPPA: Pregnancy associated plasma protein A
PE: Preeclampsia
SD: Standard deviation
wks: Weeks
