# Supplementary material for: Placental DNA methylation at term reflects maternal serum levels of INHA and FN1, but not PAPPA, early in pregnancy
Source: BMC Med Genet. 2015 Dec 11;16:111. doi: 10.1186/s12881-015-0257-z (PMC4676901; doi:10.1186/s12881-015-0257-z)
Supplement: Additional file 1: Table S1. — All samples used and the measures completed on each one. (PDF 353 kb) [file 12881_2015_257_MOESM1_ESM.pdf]

Table S1. All samples used and what measures were completed on each one.

SAMPLE ID	GROUP	450K	Gene expression	Pyrosequencing	FN1 ELISA	PAPPA (MSS)	INHA (MSS)
**PL135**	LOPE+IUGR	X					
**PL142**	LOPE+IUGR	X					
**PM31**	LOPE+IUGR	X		X	X		
**PM32**	LOPE+IUGR	X		X	X		
**PM38**	LOPE+IUGR	X		X	X		
**PM40**	LOPE+IUGR	X		X	X		
**PM52**	LOPE+IUGR	X		X	X		
**PM66**	LOPE+IUGR	X		X	X		
**PM115**	LOPE	X		X	X		
**PM119**	LOPE	X		X	X		
**PM44**	LOPE	X		X	X		
**PM46**	LOPE	X		X	X		
**PM53**	LOPE	X		X	X		
**PM54**	LOPE	X		X	X		
**PM55**	LOPE	X		X	X		
**PM58**	LOPE	X		X	X		
**PM71**	LOPE	X		X	X		
**PM98**	LOPE	X		X	X		
**PM269**	LOPE	X					
**PL145**	IUGR	X					
**PL72**	IUGR	X					
**PL86**	IUGR	X					
**PM121**	IUGR			X	X		
**PM123**	IUGR	X		X	X		
**PM128**	IUGR			X	X		
**PM130**	IUGR	X		X	X		
**PM139**	IUGR	X		X	X		
**PM29**	IUGR	X					
**PM30**	IUGR	X		X	X		
**PM35**	IUGR	X					
**PM4**	IUGR	X		X	X		
**PM41**	IUGR	X					
**PM42**	IUGR			X	X		
**PM47**	IUGR	X		X	X		
**PM72**	IUGR	X					

**PL130**	EOPE	X					
**PL131**	EOPE	X					
**PM116**	EOPE	X	X	X	X		
**PM12**	EOPE	X	X				
**PM129**	EOPE	X	X	X	X		
**PM138**	EOPE	X					
**PM15**	EOPE	X		X	X		
**PM21**	EOPE	X	X	X	X		
**PM36**	EOPE	X		X	X		
**PM39**	EOPE	X	X	X	X		
**PM43**	EOPE	X	X	X	X		
**PM49**	EOPE	X					
**PM50**	EOPE			X	X		
**PM51**	EOPE	X	X				
**PM6**	EOPE	X		X	X		
**PM60**	EOPE			X	X		
**PM62**	EOPE			X	X		
**PM64**	EOPE	X		X	X		
**PM67**	EOPE	X					
**PM80**	EOPE	X		X	X		
**PM86**	EOPE	X	X				
**PM97**	EOPE	X					
**PM99**	EOPE	X					
**PL102**	CONTROL	X					
**PL104**	CONTROL	X					
**PL11**	CONTROL	X	X				
**PL112**	CONTROL	X					
**PL113**	CONTROL	X	X				
**PL21**	CONTROL	X					
**PL25**	CONTROL	X					
**PL26**	CONTROL	X					
**PL32**	CONTROL	X					
**PL33**	CONTROL	X	X				
**PL38**	CONTROL	X					
**PL43**	CONTROL	X					
**PL56**	CONTROL	X	X				
**PL58**	CONTROL	X					
**PL59**	CONTROL	X					
**PL64**	CONTROL	X	X				
**PL65**	CONTROL	X	X				
**PL76**	CONTROL	X	X				

**PL96**	CONTROL	X	X				
**PM 266**	CONTROL			X			
**PM 268**	CONTROL			X			
**PM100**	CONTROL			X	X		
**PM101**	CONTROL			X	X		
**PM103**	CONTROL			X	X		
**PM106**	CONTROL			X	X		
**PM110**	CONTROL			X	X		
**PM111**	CONTROL			X	X		
**PM112**	CONTROL	X		X	X		
**PM113**	CONTROL			X	X		
**PM114**	CONTROL	X		X	X		
**PM117**	CONTROL			X	X		
**PM118**	CONTROL			X	X		
**PM120**	CONTROL	X		X	X		
**PM122**	CONTROL	X		X	X		
**PM124**	CONTROL			X	X		
**PM127**	CONTROL			X	X		
**PM134**	CONTROL			X	X		
**PM136**	CONTROL	X		X	X		
**PM142**	CONTROL	X					
**PM153**	CONTROL	X					
**PM158**	CONTROL	X					
**PM161**	CONTROL	X					
**PM163**	CONTROL			X	X		
**PM167**	CONTROL	X					
**PM17**	CONTROL	X					
**PM176**	CONTROL			X		X	
**PM179**	CONTROL			X	X		
**PM190**	CONTROL			X	X		X
**PM191**	CONTROL			X	X		X
**PM193**	CONTROL			X	X		X
**PM198**	CONTROL			X	X	X	
**PM20**	CONTROL	X					
**PM200**	CONTROL			X	X		X
**PM201**	CONTROL			X	X		X
**PM202**	CONTROL			X	X		
**PM205**	CONTROL	X					
**PM209**	CONTROL			X			X
**PM212**	CONTROL			X	X	X	X
**PM214**	CONTROL			X	X	X	X

**PM214-2**	CONTROL			X	X		X
**PM215**	CONTROL			X	X	X	X
**PM216**	CONTROL			X	X	X	X
**PM217**	CONTROL			X	X	X	X
**PM218**	CONTROL			X	X	X	
**PM219**	CONTROL			X	X	X	X
**PM220**	CONTROL			X	X	X	X
**PM221**	CONTROL			X	X		X
**PM222**	CONTROL			X	X	X	X
**PM223**	CONTROL			X	X	X	X
**PM224**	CONTROL			X	X	X	X
**PM227**	CONTROL			X		X	X
**PM228**	CONTROL			X	X	X	X
**PM229**	CONTROL			X	X	X	X
**PM232**	CONTROL			X	X	X	X
**PM234**	CONTROL			X	X	X	X
**PM236**	CONTROL			X		X	X
**PM237**	CONTROL			X		X	X
**PM239**	CONTROL			X	X	X	
**PM243**	CONTROL			X	X	X	X
**PM248**	CONTROL			X	X		X
**PM249**	CONTROL	X		X		X	X
**PM252**	CONTROL			X	X	X	X
**PM254**	CONTROL			X	X		X
**PM258**	CONTROL			X	X	X	X
**PM259**	CONTROL			X	X	X	X
**PM260**	CONTROL			X	X	X	X
**PM261**	CONTROL			X	X	X	
**PM262**	CONTROL			X	X	X	X
**PM263**	CONTROL	X					
**PM264**	CONTROL			X	X	X	
**PM266**	CONTROL			X	X	X	X
**PM267**	CONTROL			X	X	X	
**PM268**	CONTROL			X	X	X	X
**PM57**	CONTROL			X	X		
**PM59**	CONTROL			X	X		
**PM65**	CONTROL			X	X		
**PM70**	CONTROL			X	X		
**PM74**	CONTROL	X		X	X		
**PM77**	CONTROL	X		X	X		
**PM78**	CONTROL			X	X		

**PM79**	CONTROL			X	X		
**PM81**	CONTROL			X	X		
**PM83**	CONTROL			X	X		
**PM84**	CONTROL			X	X		
**PM85**	CONTROL			X	X		
**PM87**	CONTROL	X		X	X		
**PM88**	CONTROL			X	X		
**PM89**	CONTROL			X	X		
**PM90**	CONTROL			X	X		
**PM91**	CONTROL			X	X		
**PM93**	CONTROL			X	X		
**PM94**	CONTROL			X	X		
**PM96**	CONTROL			X	X		
