# Supplementary material for: Placental DNA methylation at term reflects maternal serum levels of INHA and FN1, but not PAPPA, early in pregnancy
Source: BMC Med Genet. 2015 Dec 11;16:111. doi: 10.1186/s12881-015-0257-z (PMC4676901; doi:10.1186/s12881-015-0257-z)
Supplement: Additional file 2: Table S2. — Information on samples used to assess DNAm and gene expression in the placenta. (PDF 174 kb) [file 12881_2015_257_MOESM2_ESM.pdf]

Table S2. Samples used to assess the relationship between DNAm and gene expression in the term placenta.

	**Control**	**EOPE**
**N=**	8	8
Mean GA at delivery (weeks ±SD)	31.5 (±3.9)	32.2 (±3.9)
Mean BW (grams ±SD)	1885.0 (±677.2)	1420.0 (±394.8)
Mean MA (years ±SD)	30.2 (±5.9)	33.4 (±5.9)
Sex (Female/N, %)	2/8, 25%	2/8, 25%

† Previously published in Blair *et al.* 2013.
