# Supplementary material for: Placental DNA methylation at term reflects maternal serum levels of INHA and FN1, but not PAPPA, early in pregnancy
Source: BMC Med Genet. 2015 Dec 11;16:111. doi: 10.1186/s12881-015-0257-z (PMC4676901; doi:10.1186/s12881-015-0257-z)
Supplement: Additional file 3: Table S3. — Primer sequences for bisulfite pyrosequencing. Specific locations are based on UCSC hg/18 assembly. (PDF 296 kb) [file 12881_2015_257_MOESM3_ESM.pdf]

Table S3. Primer sequences for bisulfite pyrosequencing. Specific locations are based on UCSC hg/18 assembly.

Gene	Primer	Sequence	Genomic Target	PCR conditions	DNA loaded into Pyro
INHBA	Forward	GGAGTGGGAGATAAGGTTTATGGTTTA	Chr2: 220436857-220436999	95(15')/95(30"), **55**(30"), 72(30") x40/72(10')	5uL
	Reverse	ACACCCACCCTCTTCTACCCT			
	Sequencing	GGTTTATGGTTATAGATATT			
		CpG 1	Chr2: 220436894		
PAPPA	Forward	GGGATTTATTTAATTGGGAAGGTG	Chr9: 118915879-118915998	95(15')/95(30"), **55**(30"), 72(30") x40/72(10')	8uL
	Reverse	CCTCCCTCCTTCTTCTCAAA			
	Sequencing	TTAATTGGGAAGGTGG			
		CpG 1	118915907		
		CpG 2	118915929		
FN1	Forward	TGGGGGAAAFAFFTTATAGATAT	Chr 2: 216402317-216402556	95(15')/95(30"), **55**(30"), 72(30") x40/72(10')	8uL
	Reverse	ACAAAAATTACCAAATCCTAAACATTTACT			
	Sequencing	TGAGTTGTATTTTTTGGAAGT			
		CpG 1	216402384		
