# Supplementary material for: Placental DNA methylation at term reflects maternal serum levels of INHA and FN1, but not PAPPA, early in pregnancy
Source: BMC Med Genet. 2015 Dec 11;16:111. doi: 10.1186/s12881-015-0257-z (PMC4676901; doi:10.1186/s12881-015-0257-z)
Supplement: Additional file 4: Figure S1. — Spearman’s correlation between CpG 1 and CpG 2 within the PAPPA assay. DNA methylation was averaged over the two CpGs (PDF 249 kb) [file 12881_2015_257_MOESM4_ESM.pdf]

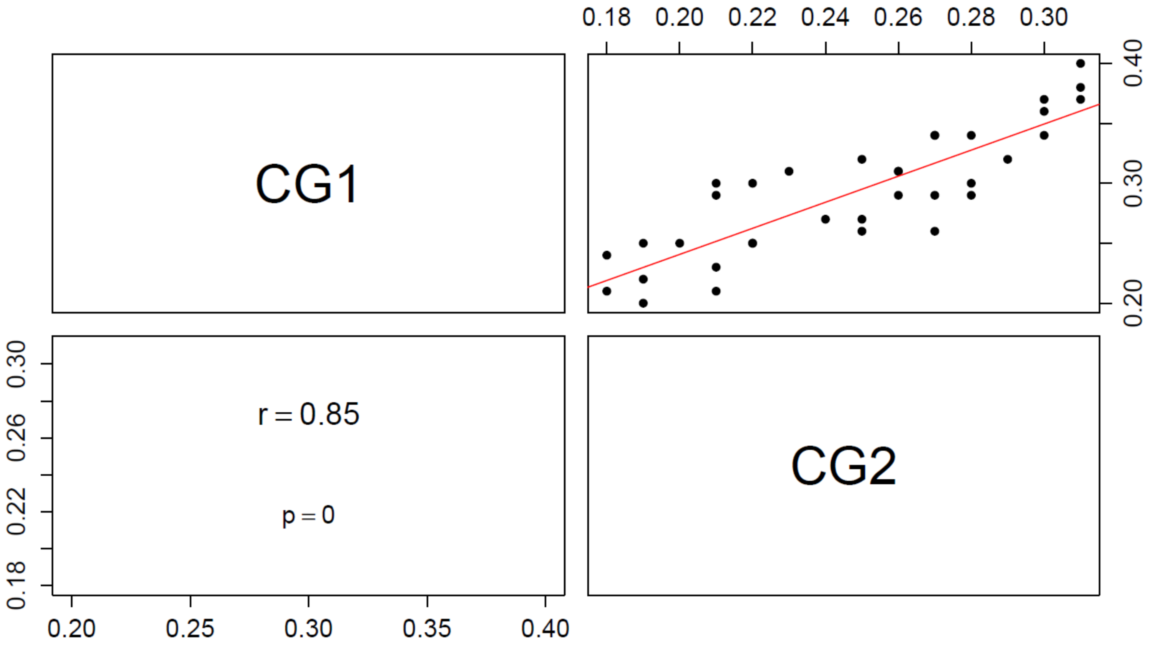

Figure S1. A Spearman's correlation was performed between CpG 1 and CpG 2 within the *PAPPA* assay. DNA methylation was averaged over the two CpGs
